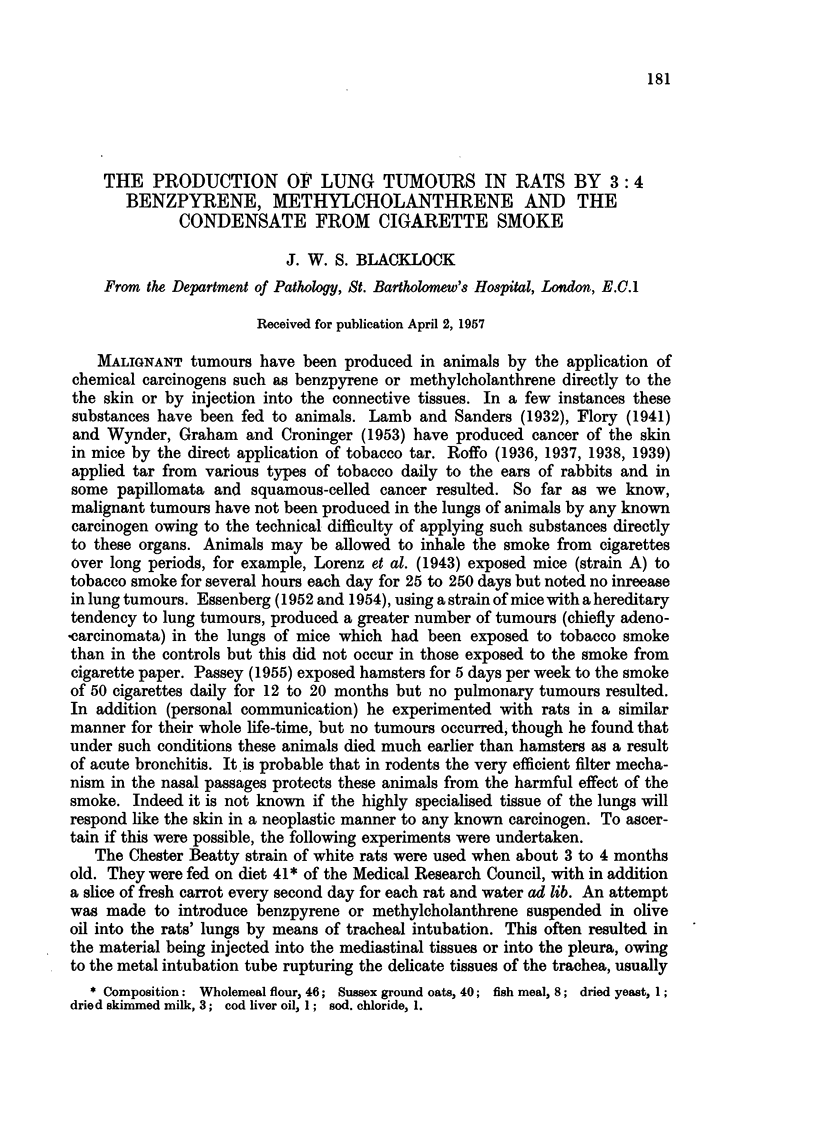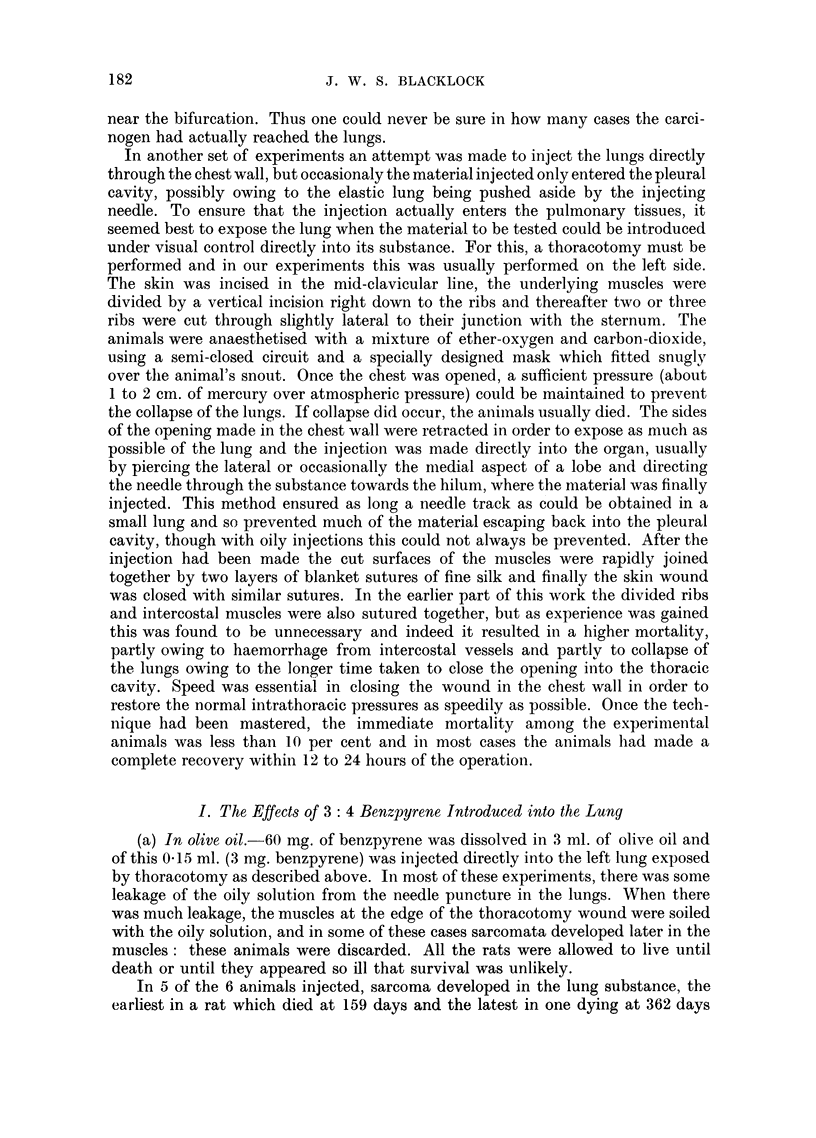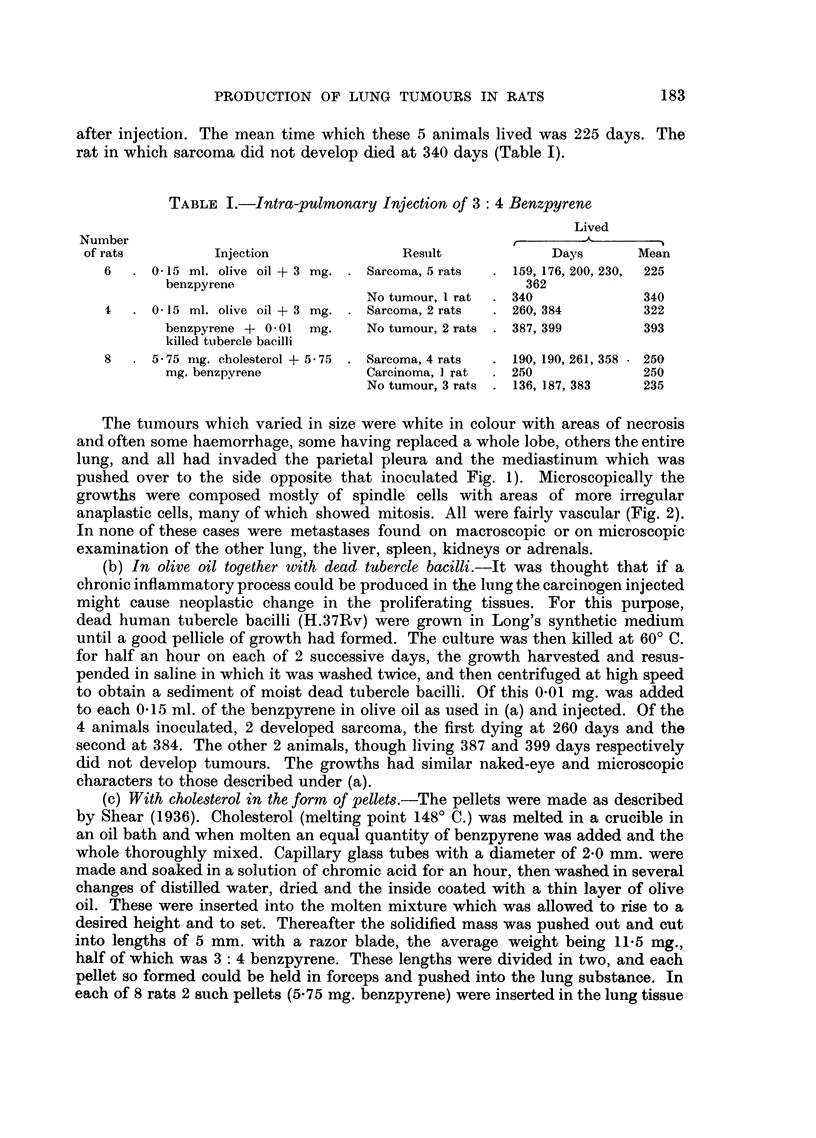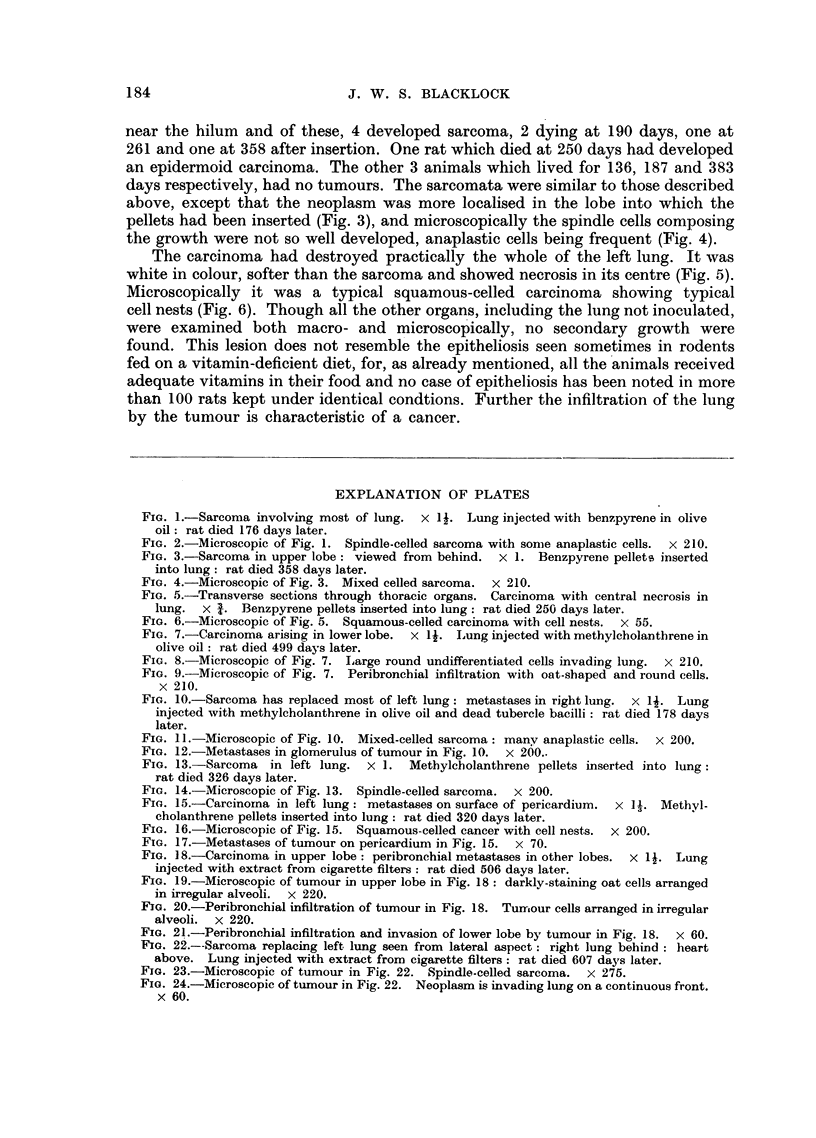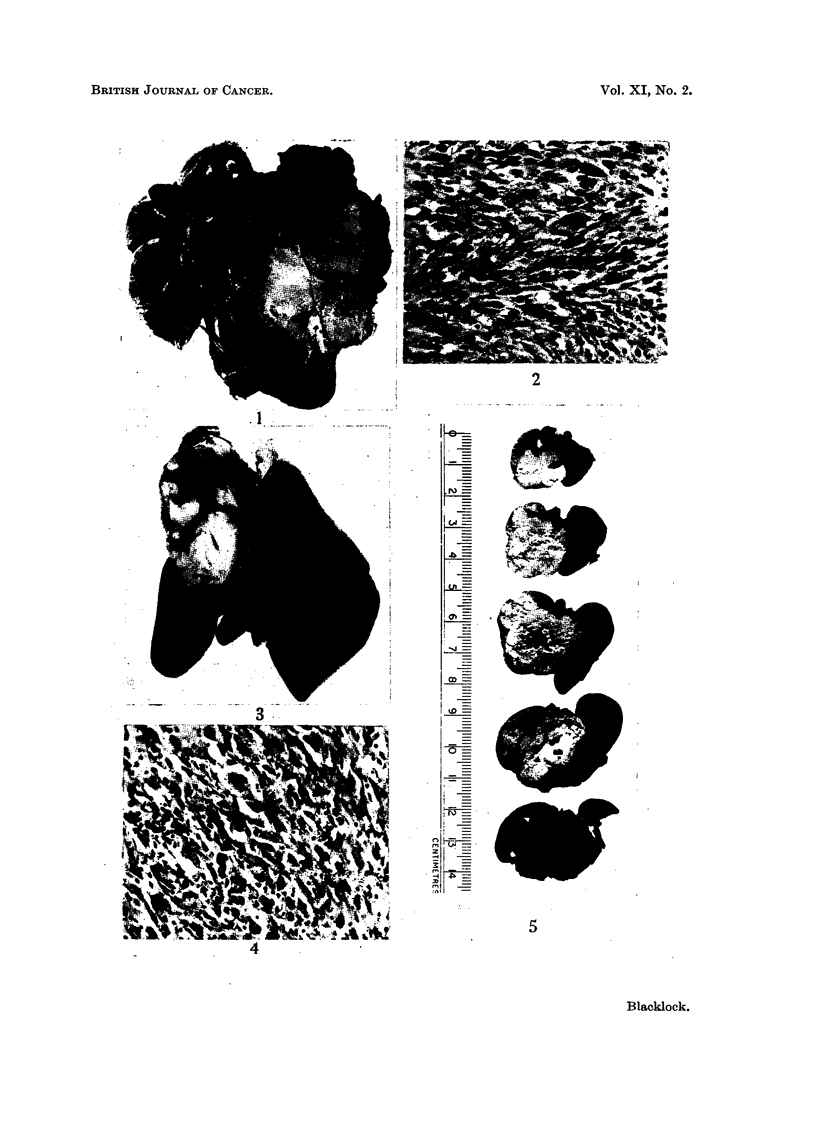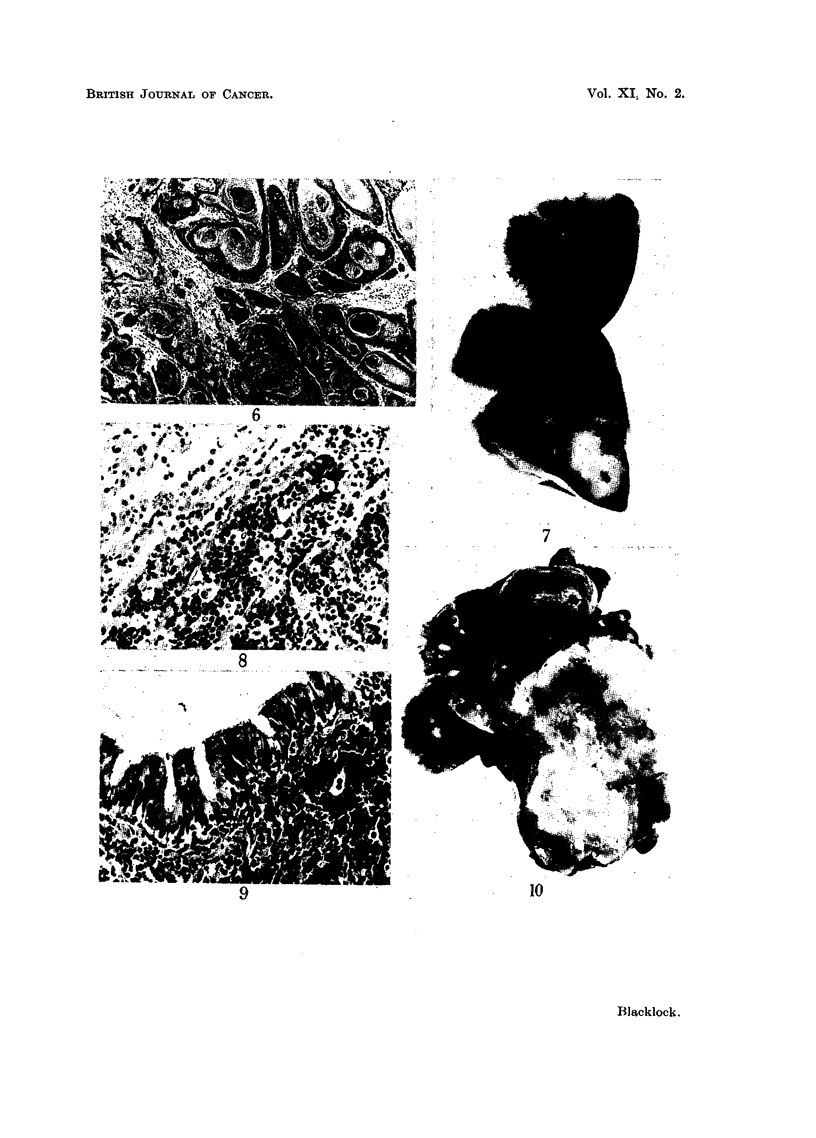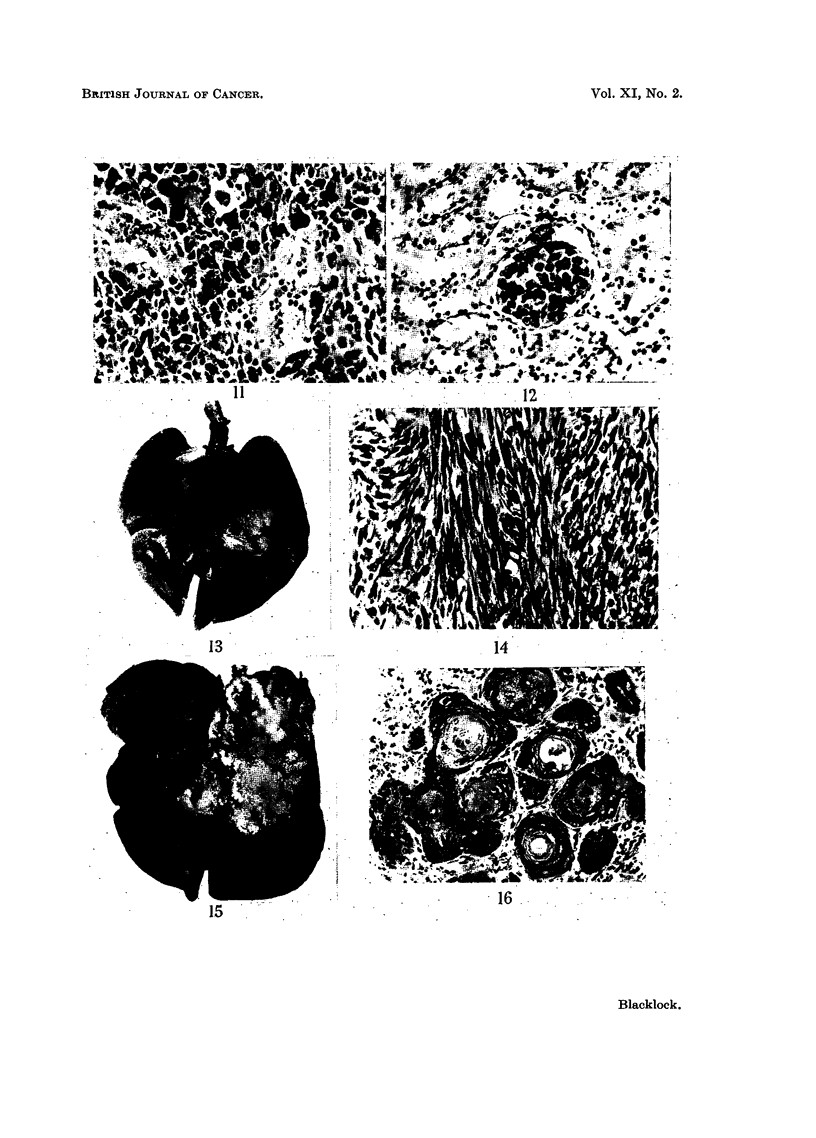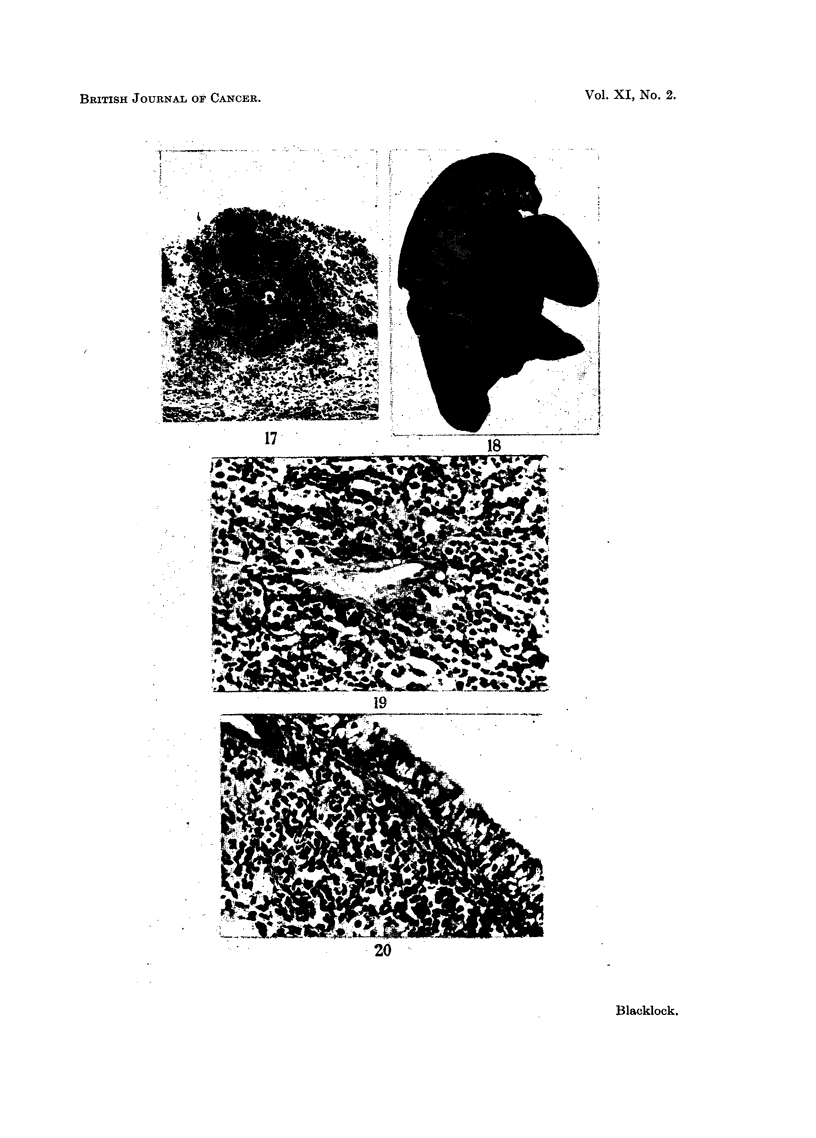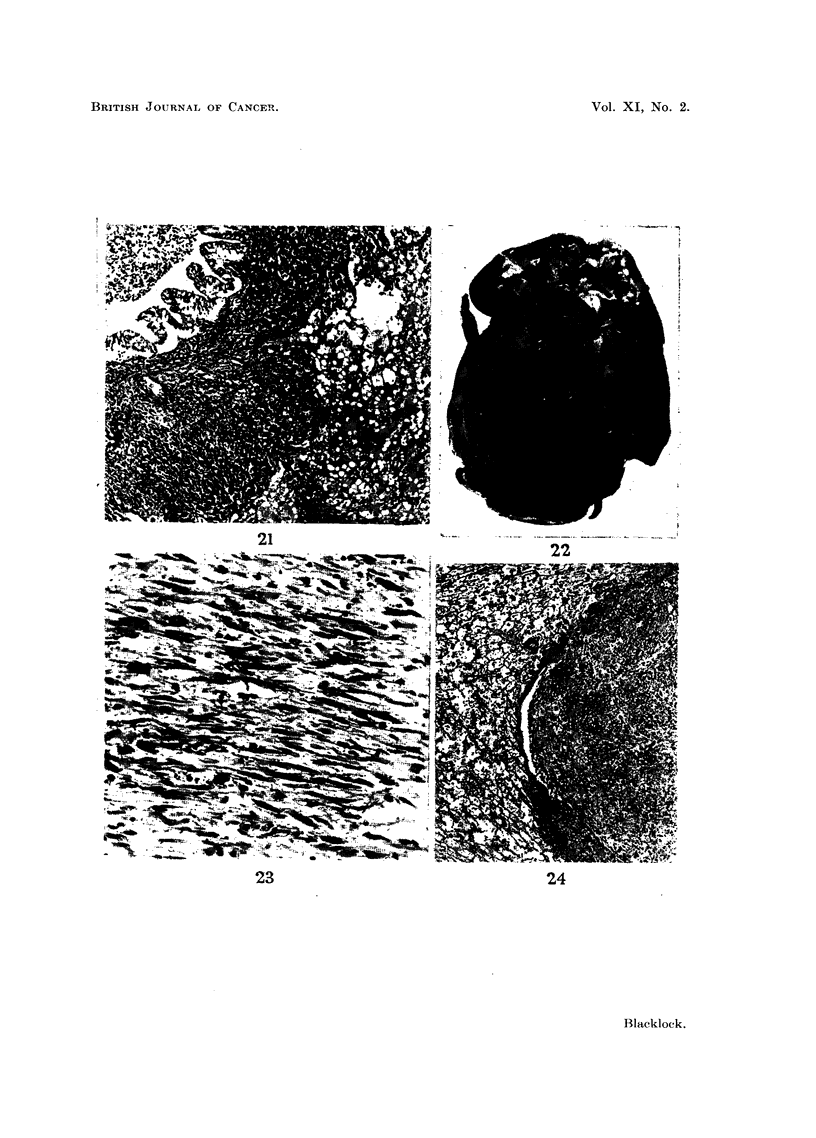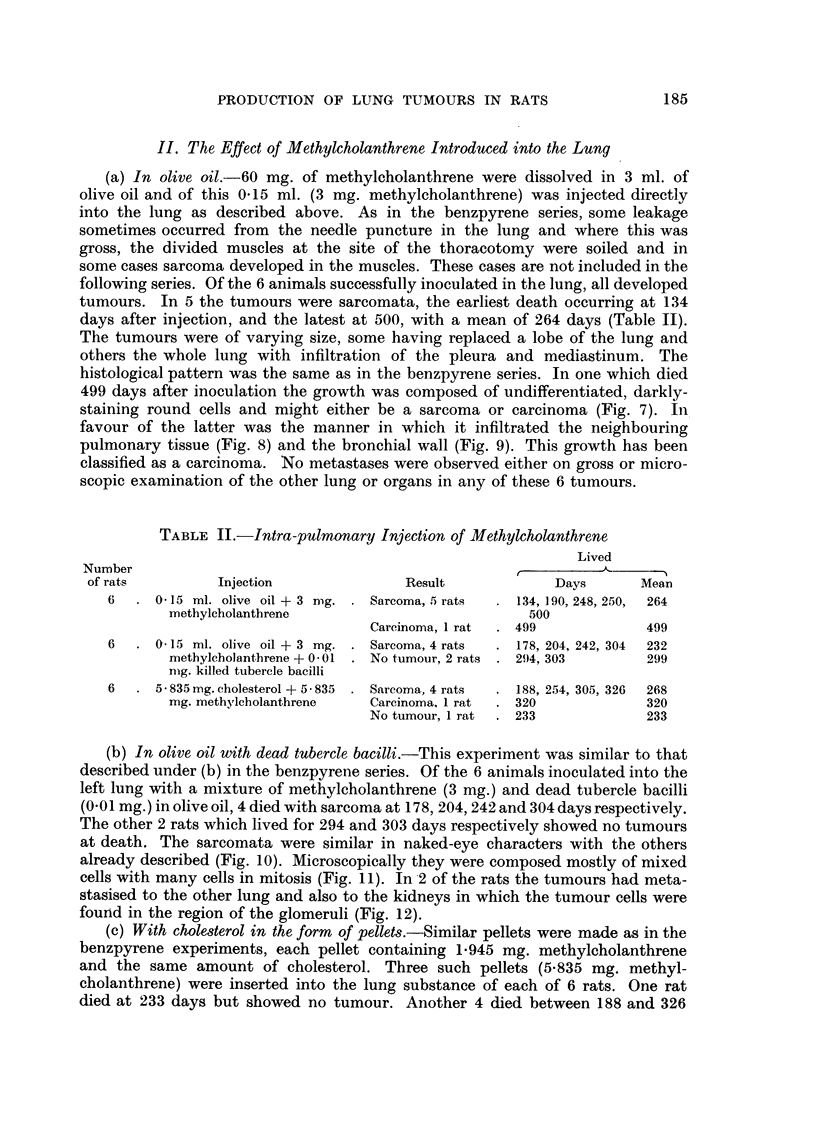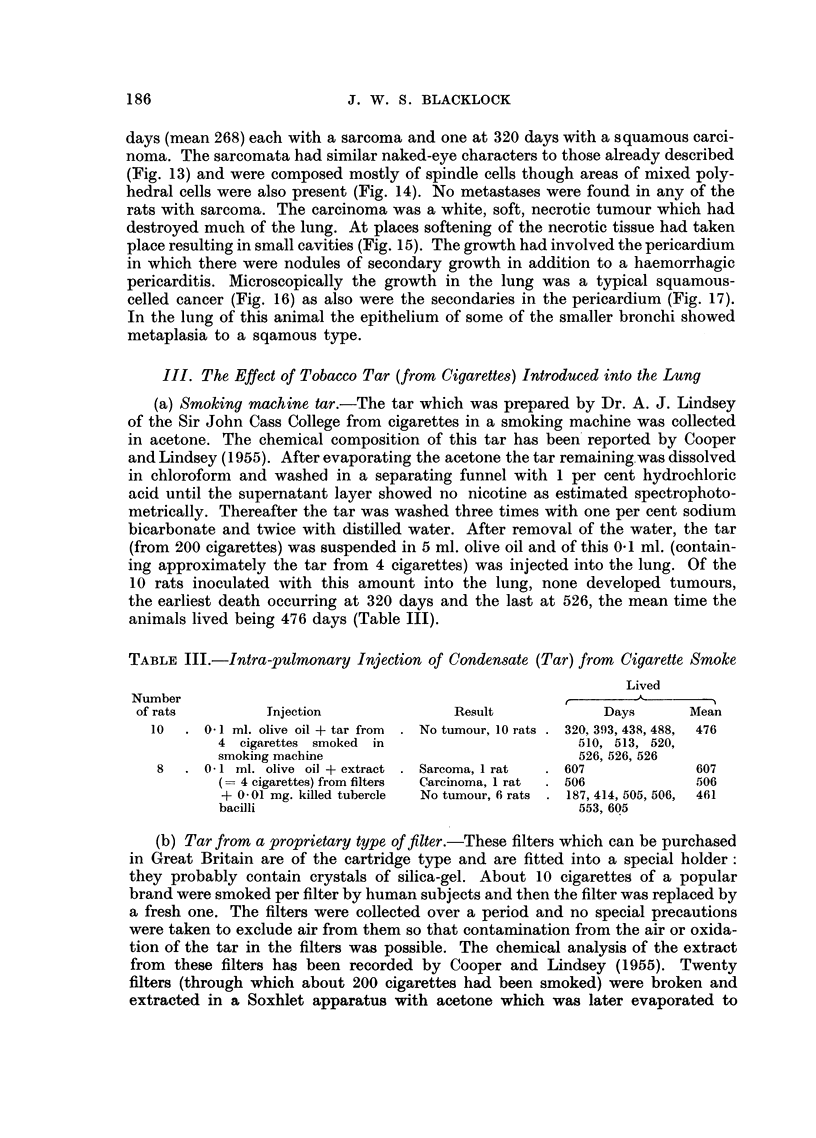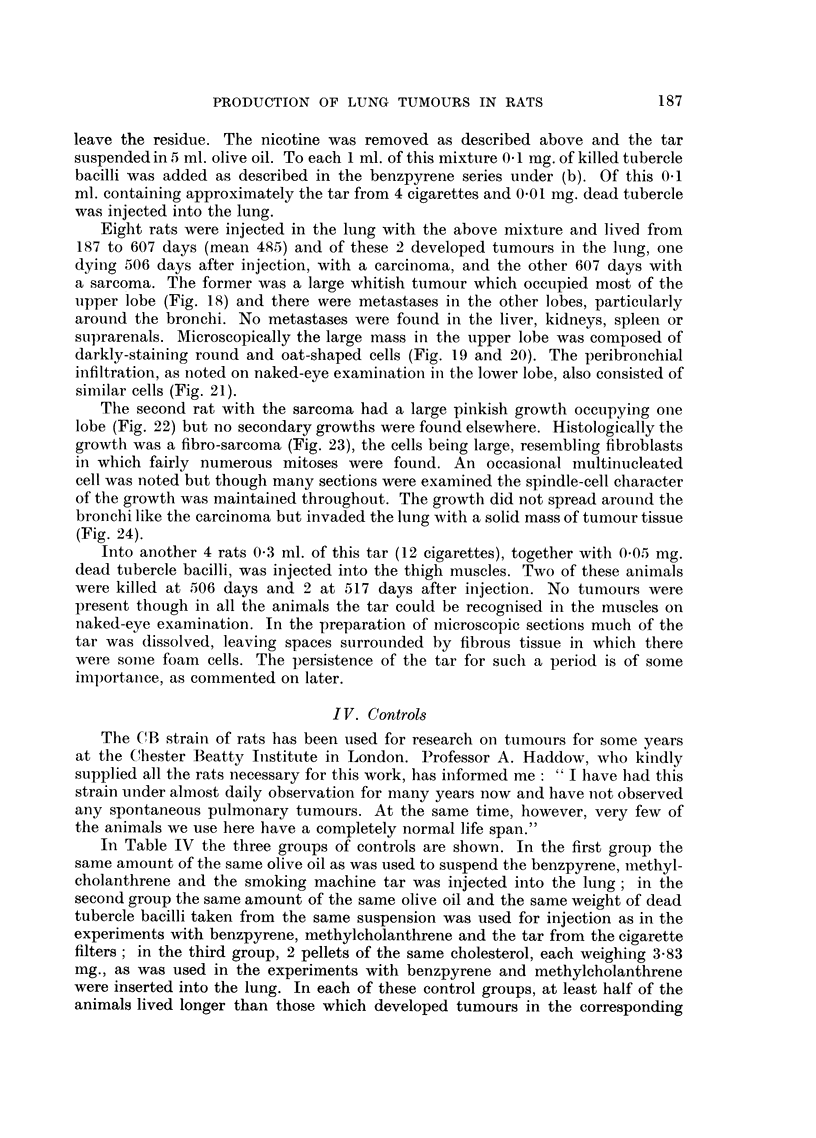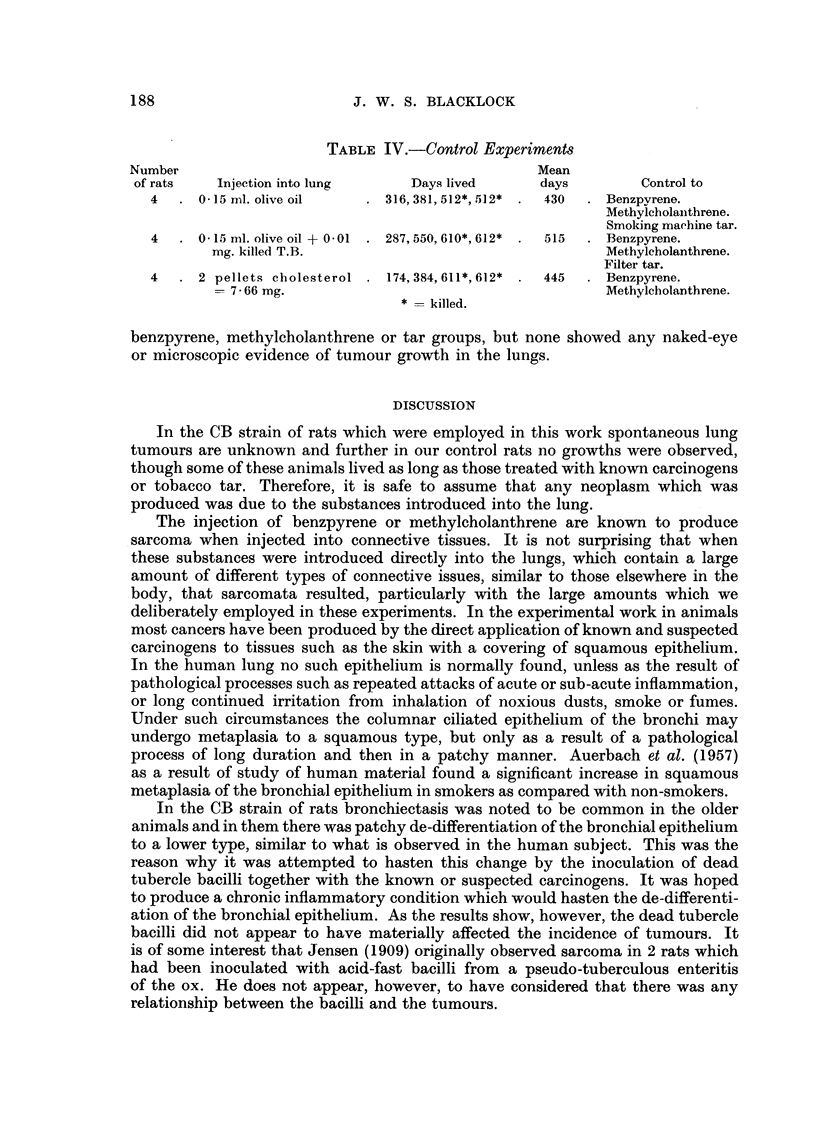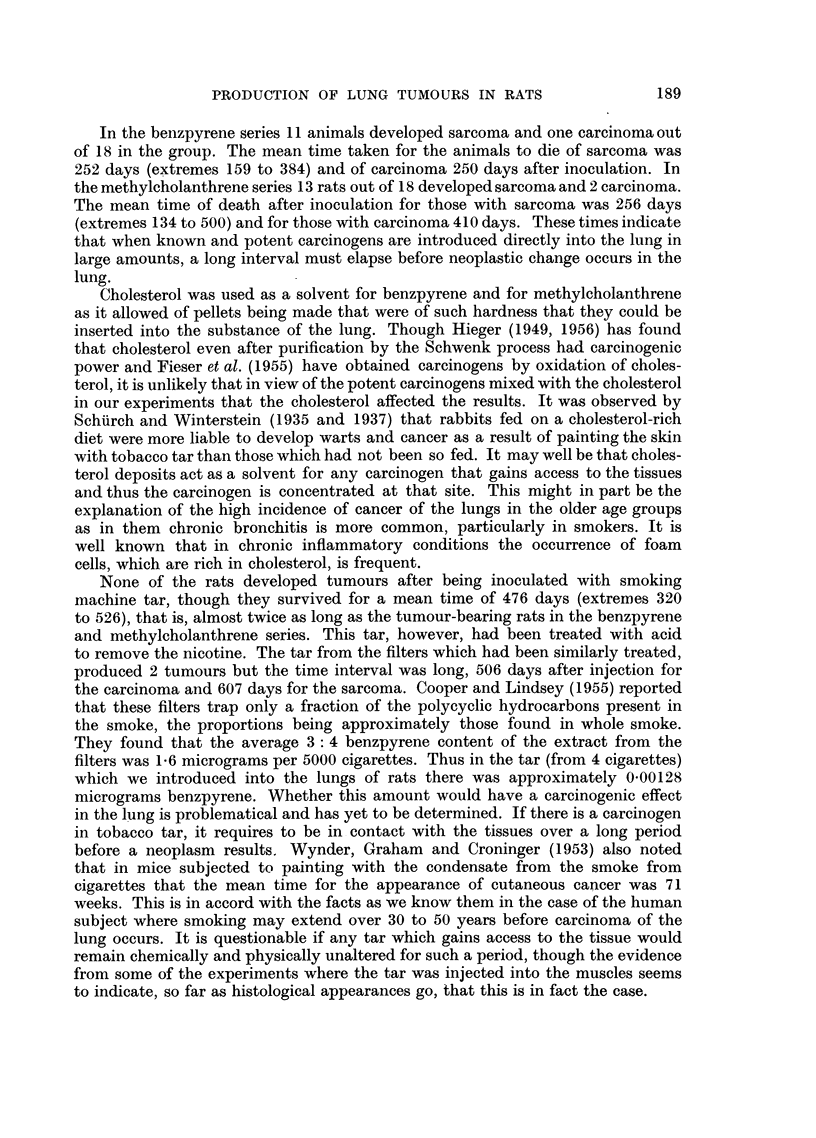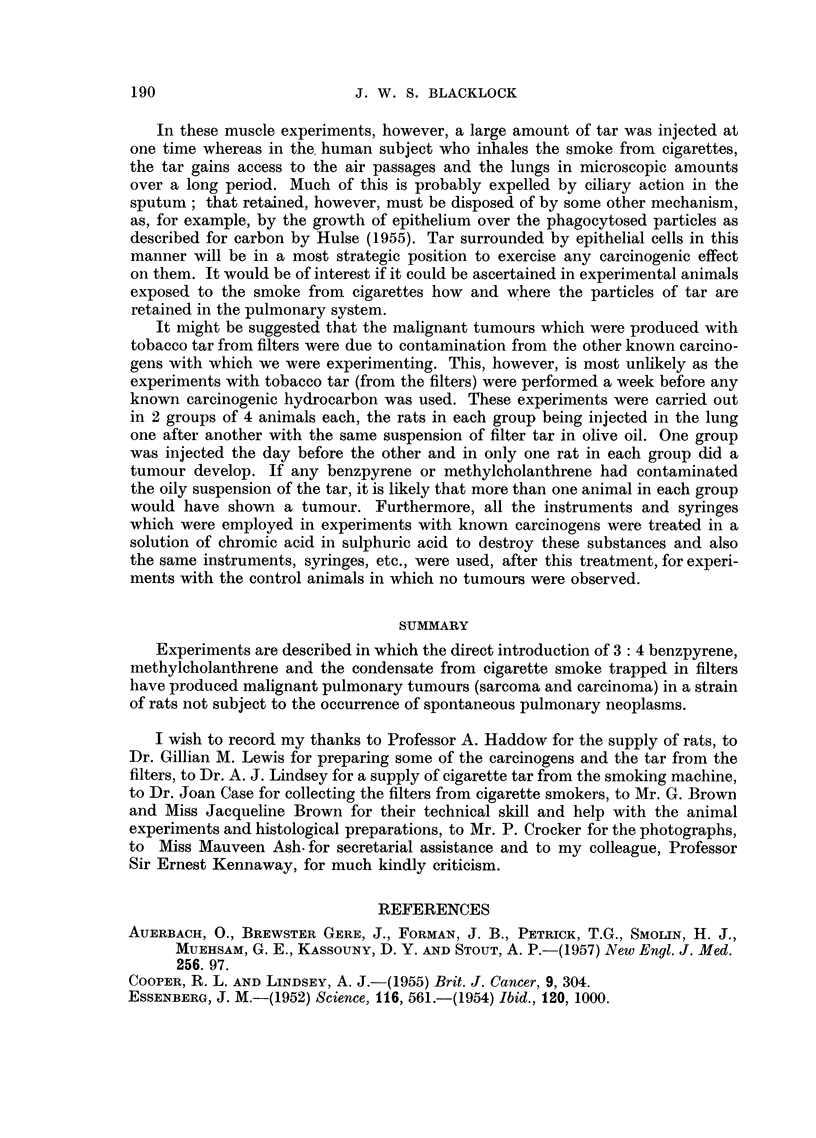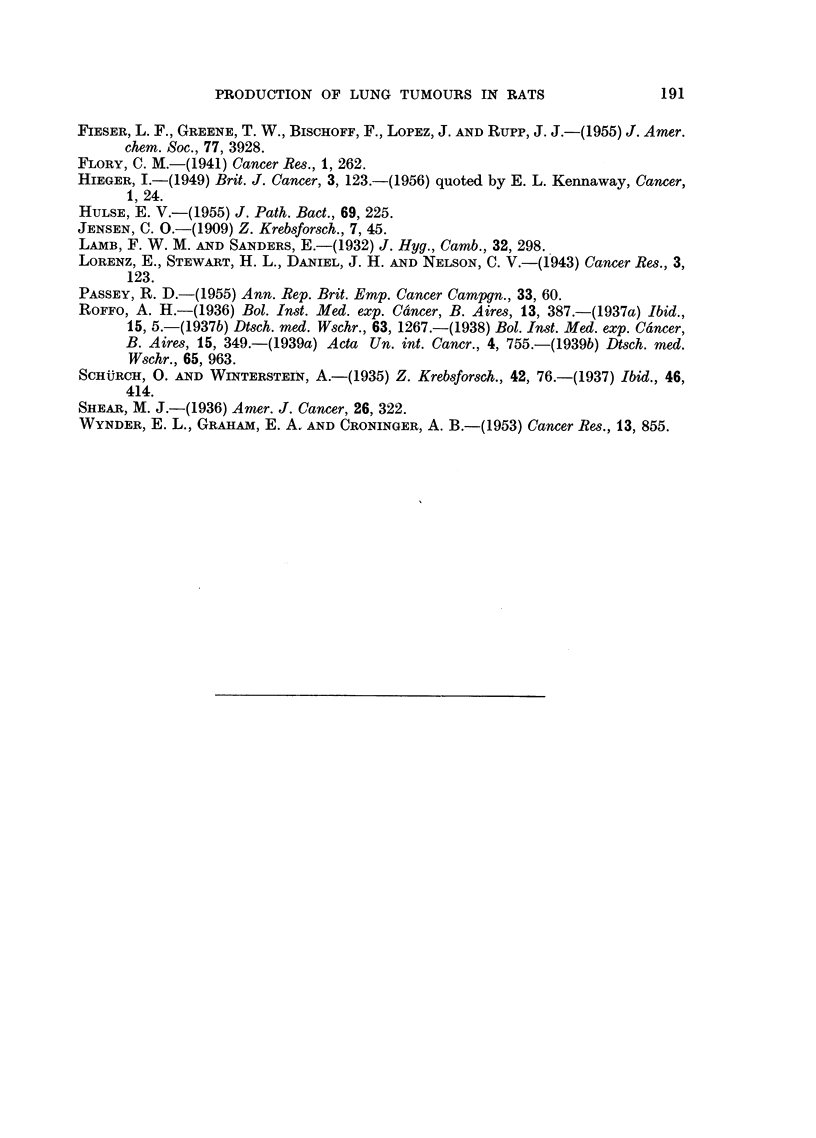# The Production of Lung Tumours in Rats by 3:4 Benzpyrene, Methylcholanthrene and the Condensate from Cigarette Smoke

**DOI:** 10.1038/bjc.1957.25

**Published:** 1957-06

**Authors:** J. W. S. Blacklock

## Abstract

**Images:**


### THE PRODUCTION OF LUNG TUMOURS IN RATS BY 3 : 4 BENZPYRENE, METHYLCHOLANTHRENE AND THE CONDENSATE FROM CIGARETTE SMOKE

J. W. S. BLACKLOCK

*From the Department of Pathology, St. Bartholomew's Hospital, London, E.C.*1

Received for publication April 2, 1957

MALIGNANT tumours have been produced in animals by the application of chemical carcinogens such as benzpyrene or methylcholanthrene directly to the the skin or by injection into the connective tissues. In a few instances these substances have been fed to animals. Lamb and Sanders (1932), Flory (1941) and Wynder, Graham and Croninger (1953) have produced cancer of the skin in mice by the direct application of tobacco tar. Roffo (1936, 1937, 1938, 1939) applied tar from various types of tobacco daily to the ears of rabbits and in some papillomata and squamous-celled cancer resulted. So far as we know, malignant tumours have not been produced in the lungs of animals by any known carcinogen owing to the technical difficulty of applying such substances directly to these organs. Animals may be allowed to inhale the smoke from cigarettes over long periods, for example, Lorenz *et al.* (1943) exposed mice (strain A) to tobacco smoke for several hours each day for 25 to 250 days but noted no inreease in lung tumours. Essenberg (1952 and 1954), using a strain of mice with a hereditary tendency to lung tumours, produced a greater number of tumours (chiefly adeno-carcinomata) in the lungs of mice which had been exposed to tobacco smoke than in the controls but this did not occur in those exposed to the smoke from cigarette paper. Passey (1955) exposed hamsters for 5 days per week to the smoke of 50 cigarettes daily for 12 to 20 months but no pulmonary tumours resulted. In addition (personal communication) he experimented with rats in a similar manner for their whole life-time, but no tumours occurred, though he found that under such conditions these animals died much earlier than hamsters as a result of acute bronchitis. It is probable that in rodents the very efficient filter mechanism in the nasal passages protects these animals from the harmful effect of the smoke. Indeed it is not known if the highly specialised tissue of the lungs will respond like the skin in a neoplastic manner to any known carcinogen. To ascertain if this were possible, the following experiments were undertaken.

The Chester Beatty strain of white rats were used when about 3 to 4 months old. They were fed on diet 41* of the Medical Research Council, with in addition a slice of fresh carrot every second day for each rat and water *ad lib.* An attempt was made to introduce benzpyrene or methylcholanthrene suspended in olive oil into the rats' lungs by means of tracheal intubation. This often resulted in the material being injected into the mediastinal tissues or into the pleura, owing to the metal intubation tube rupturing the delicate tissues of the trachea, usually

* Composition: Wholemeal flour, 46; Sussex ground oats, 40; fish meal, 8; dried yeast, 1; dried skimmed milk, 3; cod liver oil, 1; sod. chloride, 1.

near the bifurcation. Thus one could never be sure in how many cases the carcinogen had actually reached the lungs.

In another set of experiments an attempt was made to inject the lungs directly through the chest wall, but occasionaly the material injected only entered the pleural cavity, possibly owing to the elastic lung being pushed aside by the injecting needle. To ensure that the injection actually enters the pulmonary tissues, it seemed best to expose the lung when the material to be tested could be introduced under visual control directly into its substance. For this, a thoracotomy must be performed and in our experiments this was usually performed on the left side. The skin was incised in the mid-clavicular line, the underlying muscles were divided by a vertical incision right down to the ribs and thereafter two or three ribs were cut through slightly lateral to their junction with the sternum. The animals were anaesthetised with a mixture of ether-oxygen and carbon-dioxide, using a semi-closed circuit and a specially designed mask which fitted snugly over the animal's snout. Once the chest was opened, a sufficient pressure (about 1 to 2 cm. of mercury over atmospheric pressure) could be maintained to prevent the collapse of the lungs. If collapse did occur, the animals usually died. The sides of the opening made in the chest wall were retracted in order to expose as much as possible of the lung and the injection was made directly into the organ, usually by piercing the lateral or occasionally the medial aspect of a lobe and directing the needle through the substance towards the hilum, where the material was finally injected. This method ensured as long a needle track as could be obtained in a small lung and so prevented much of the material escaping back into the pleural cavity, though with oily injections this could not always be prevented. After the injection had been made the cut surfaces of the muscles were rapidly joined together by two layers of blanket sutures of fine silk and finally the skin wound was closed with similar sutures. In the earlier part of this work the divided ribs and intercostal muscles were also sutured together, but as experience was gained this was found to be unnecessary and indeed it resulted in a higher mortality, partly owing to haemorrhage from intercostal vessels and partly to collapse of the lungs owing to the longer time taken to close the opening into the thoracic cavity. Speed was essential in closing the wound in the chest wall in order to restore the normal intrathoracic pressures as speedily as possible. Once the technique had been mastered, the immediate mortality among the experimental animals was less than 10 per cent and in most cases the animals had made a complete recovery within 12 to 24 hours of the operation.

### *I. The Effects of* 3 : 4 *Benzpyrene Introduced into the Lung*

(a) *In olive oil.*—60 mg. of benzpyrene was dissolved in 3 ml. of olive oil and of this 0·15 ml. (3 mg. benzpyrene) was injected directly into the left lung exposed by thoracotomy as described above. In most of these experiments, there was some leakage of the oily solution from the needle puncture in the lungs. When there was much leakage, the muscles at the edge of the thoracotomy wound were soiled with the oily solution, and in some of these cases sarcomata developed later in the muscles : these animals were discarded. All the rats were allowed to live until death or until they appeared so ill that survival was unlikely.

In 5 of the 6 animals injected, sarcoma developed in the lung substance, the earliest in a rat which died at 159 days and the latest in one dying at 362 days after injection. The mean time which these 5 animals lived was 225 days. The rat in which sarcoma did not develop died at 340 days (Table I).

TABLE I.—*Intra-pulmonary Injection of* 3 : 4 *Benzpyrene*

| Number of rats | Injection | Result | Lived: Days | Lived: Mean |
|---|---|---|---|---|
| 6 | 0·15 ml. olive oil + 3 mg. benzpyrene | Sarcoma, 5 rats | 159, 176, 200, 230, 362 | 225 |
| | | No tumour, 1 rat | 340 | 340 |
| 4 | 0·15 ml. olive oil + 3 mg. benzpyrene + 0·01 mg. killed tubercle bacilli | Sarcoma, 2 rats | 260, 384 | 322 |
| | | No tumour, 2 rats | 387, 399 | 393 |
| 8 | 5·75 mg. cholesterol + 5·75 mg. benzpyrene | Sarcoma, 4 rats | 190, 190, 261, 358 | 250 |
| | | Carcinoma, 1 rat | 250 | 250 |
| | | No tumour, 3 rats | 136, 187, 383 | 235 |

The tumours which varied in size were white in colour with areas of necrosis and often some haemorrhage, some having replaced a whole lobe, others the entire lung, and all had invaded the parietal pleura and the mediastinum which was pushed over to the side opposite that inoculated Fig. 1). Microscopically the growths were composed mostly of spindle cells with areas of more irregular anaplastic cells, many of which showed mitosis. All were fairly vascular (Fig. 2). In none of these cases were metastases found on macroscopic or on microscopic examination of the other lung, the liver, spleen, kidneys or adrenals.

(b) *In olive oil together with dead tubercle bacilli.*—It was thought that if a chronic inflammatory process could be produced in the lung the carcinogen injected might cause neoplastic change in the proliferating tissues. For this purpose, dead human tubercle bacilli (H.37Rv) were grown in Long's synthetic medium until a good pellicle of growth had formed. The culture was then killed at 60° C. for half an hour on each of 2 successive days, the growth harvested and resuspended in saline in which it was washed twice, and then centrifuged at high speed to obtain a sediment of moist dead tubercle bacilli. Of this 0·01 mg. was added to each 0·15 ml. of the benzpyrene in olive oil as used in (a) and injected. Of the 4 animals inoculated, 2 developed sarcoma, the first dying at 260 days and the second at 384. The other 2 animals, though living 387 and 399 days respectively did not develop tumours. The growths had similar naked-eye and microscopic characters to those described under (a).

(c) *With cholesterol in the form of pellets.*—The pellets were made as described by Shear (1936). Cholesterol (melting point 148° C.) was melted in a crucible in an oil bath and when molten an equal quantity of benzpyrene was added and the whole thoroughly mixed. Capillary glass tubes with a diameter of 2·0 mm. were made and soaked in a solution of chromic acid for an hour, then washed in several changes of distilled water, dried and the inside coated with a thin layer of olive oil. These were inserted into the molten mixture which was allowed to rise to a desired height and to set. Thereafter the solidified mass was pushed out and cut into lengths of 5 mm. with a razor blade, the average weight being 11·5 mg., half of which was 3 : 4 benzpyrene. These lengths were divided in two, and each pellet so formed could be held in forceps and pushed into the lung substance. In each of 8 rats 2 such pellets (5·75 mg. benzpyrene) were inserted in the lung tissue near the hilum and of these, 4 developed sarcoma, 2 dying at 190 days, one at 261 and one at 358 after insertion. One rat which died at 250 days had developed an epidermoid carcinoma. The other 3 animals which lived for 136, 187 and 383 days respectively, had no tumours. The sarcomata were similar to those described above, except that the neoplasm was more localised in the lobe into which the pellets had been inserted (Fig. 3), and microscopically the spindle cells composing the growth were not so well developed, anaplastic cells being frequent (Fig. 4).

The carcinoma had destroyed practically the whole of the left lung. It was white in colour, softer than the sarcoma and showed necrosis in its centre (Fig. 5). Microscopically it was a typical squamous-celled carcinoma showing typical cell nests (Fig. 6). Though all the other organs, including the lung not inoculated, were examined both macro- and microscopically, no secondary growth were found. This lesion does not resemble the epitheliosis seen sometimes in rodents fed on a vitamin-deficient diet, for, as already mentioned, all the animals received adequate vitamins in their food and no case of epitheliosis has been noted in more than 100 rats kept under identical condtions. Further the infiltration of the lung by the tumour is characteristic of a cancer.

---

EXPLANATION OF PLATES

FIG. 1.—Sarcoma involving most of lung. × 1½. Lung injected with benzpyrene in olive oil : rat died 176 days later.
FIG. 2.—Microscopic of Fig. 1. Spindle-celled sarcoma with some anaplastic cells. × 210.
FIG. 3.—Sarcoma in upper lobe : viewed from behind. × 1. Benzpyrene pellets inserted into lung : rat died 358 days later.
FIG. 4.—Microscopic of Fig. 3. Mixed celled sarcoma. × 210.
FIG. 5.—Transverse sections through thoracic organs. Carcinoma with central necrosis in lung. × ¾. Benzpyrene pellets inserted into lung : rat died 250 days later.
FIG. 6.—Microscopic of Fig. 5. Squamous-celled carcinoma with cell nests. × 55.
FIG. 7.—Carcinoma arising in lower lobe. × 1½. Lung injected with methylcholanthrene in olive oil : rat died 499 days later.
FIG. 8.—Microscopic of Fig. 7. Large round undifferentiated cells invading lung. × 210.
FIG. 9.—Microscopic of Fig. 7. Peribronchial infiltration with oat-shaped and round cells. × 210.
FIG. 10.—Sarcoma has replaced most of left lung : metastases in right lung. × 1½. Lung injected with methylcholanthrene in olive oil and dead tubercle bacilli : rat died 178 days later.
FIG. 11.—Microscopic of Fig. 10. Mixed-celled sarcoma : many anaplastic cells. × 200.
FIG. 12.—Metastases in glomerulus of tumour in Fig. 10. × 200.
FIG. 13.—Sarcoma in left lung. × 1. Methylcholanthrene pellets inserted into lung : rat died 326 days later.
FIG. 14.—Microscopic of Fig. 13. Spindle-celled sarcoma. × 200.
FIG. 15.—Carcinoma in left lung : metastases on surface of pericardium. × 1⅓. Methylcholanthrene pellets inserted into lung : rat died 320 days later.
FIG. 16.—Microscopic of Fig. 15. Squamous-celled cancer with cell nests. × 200.
FIG. 17.—Metastases of tumour on pericardium in Fig. 15. × 70.
FIG. 18.—Carcinoma in upper lobe : peribronchial metastases in other lobes. × 1½. Lung injected with extract from cigarette filters : rat died 506 days later.
FIG. 19.—Microscopic of tumour in upper lobe in Fig. 18 : darkly-staining oat cells arranged in irregular alveoli. × 220.
FIG. 20.—Peribronchial infiltration of tumour in Fig. 18. Tumour cells arranged in irregular alveoli. × 220.
FIG. 21.—Peribronchial infiltration and invasion of lower lobe by tumour in Fig. 18. × 60.
FIG. 22.—Sarcoma replacing left lung seen from lateral aspect : right lung behind : heart above. Lung injected with extract from cigarette filters : rat died 607 days later.
FIG. 23.—Microscopic of tumour in Fig. 22. Spindle-celled sarcoma. × 275.
FIG. 24.—Microscopic of tumour in Fig. 22. Neoplasm is invading lung on a continuous front. × 60.

1

2

3

4

5

Blacklock.

6

7

8

9

10

11

12

13

14

15

16

17

18

19

20

21

22

23

24

Blacklock.

### *II. The Effect of Methylcholanthrene Introduced into the Lung*

(a) *In olive oil.*—60 mg. of methylcholanthrene were dissolved in 3 ml. of olive oil and of this 0·15 ml. (3 mg. methylcholanthrene) was injected directly into the lung as described above. As in the benzpyrene series, some leakage sometimes occurred from the needle puncture in the lung and where this was gross, the divided muscles at the site of the thoracotomy were soiled and in some cases sarcoma developed in the muscles. These cases are not included in the following series. Of the 6 animals successfully inoculated in the lung, all developed tumours. In 5 the tumours were sarcomata, the earliest death occurring at 134 days after injection, and the latest at 500, with a mean of 264 days (Table II). The tumours were of varying size, some having replaced a lobe of the lung and others the whole lung with infiltration of the pleura and mediastinum. The histological pattern was the same as in the benzpyrene series. In one which died 499 days after inoculation the growth was composed of undifferentiated, darkly-staining round cells and might either be a sarcoma or carcinoma (Fig. 7). In favour of the latter was the manner in which it infiltrated the neighbouring pulmonary tissue (Fig. 8) and the bronchial wall (Fig. 9). This growth has been classified as a carcinoma. No metastases were observed either on gross or microscopic examination of the other lung or organs in any of these 6 tumours.

TABLE II.—*Intra-pulmonary Injection of Methylcholanthrene*

| Number of rats | Injection | Result | Lived: Days | Lived: Mean |
|---|---|---|---|---|
| 6 | 0·15 ml. olive oil + 3 mg. methylcholanthrene | Sarcoma, 5 rats | 134, 190, 248, 250, 500 | 264 |
| | | Carcinoma, 1 rat | 499 | 499 |
| 6 | 0·15 ml. olive oil + 3 mg. methylcholanthrene + 0·01 mg. killed tubercle bacilli | Sarcoma, 4 rats | 178, 204, 242, 304 | 232 |
| | | No tumour, 2 rats | 294, 303 | 299 |
| 6 | 5·835 mg. cholesterol + 5·835 mg. methylcholanthrene | Sarcoma, 4 rats | 188, 254, 305, 326 | 268 |
| | | Carcinoma, 1 rat | 320 | 320 |
| | | No tumour, 1 rat | 233 | 233 |

(b) *In olive oil with dead tubercle bacilli.*—This experiment was similar to that described under (b) in the benzpyrene series. Of the 6 animals inoculated into the left lung with a mixture of methylcholanthrene (3 mg.) and dead tubercle bacilli (0·01 mg.) in olive oil, 4 died with sarcoma at 178, 204, 242 and 304 days respectively. The other 2 rats which lived for 294 and 303 days respectively showed no tumours at death. The sarcomata were similar in naked-eye characters with the others already described (Fig. 10). Microscopically they were composed mostly of mixed cells with many cells in mitosis (Fig. 11). In 2 of the rats the tumours had metastasised to the other lung and also to the kidneys in which the tumour cells were found in the region of the glomeruli (Fig. 12).

(c) *With cholesterol in the form of pellets.*—Similar pellets were made as in the benzpyrene experiments, each pellet containing 1·945 mg. methylcholanthrene and the same amount of cholesterol. Three such pellets (5·835 mg. methylcholanthrene) were inserted into the lung substance of each of 6 rats. One rat died at 233 days but showed no tumour. Another 4 died between 188 and 326

days (mean 268) each with a sarcoma and one at 320 days with a squamous carcinoma. The sarcomata had similar naked-eye characters to those already described (Fig. 13) and were composed mostly of spindle cells though areas of mixed polyhedral cells were also present (Fig. 14). No metastases were found in any of the rats with sarcoma. The carcinoma was a white, soft, necrotic tumour which had destroyed much of the lung. At places softening of the necrotic tissue had taken place resulting in small cavities (Fig. 15). The growth had involved the pericardium in which there were nodules of secondary growth in addition to a haemorrhagic pericarditis. Microscopically the growth in the lung was a typical squamous-celled cancer (Fig. 16) as also were the secondaries in the pericardium (Fig. 17). In the lung of this animal the epithelium of some of the smaller bronchi showed metaplasia to a sqamous type.

### *III. The Effect of Tobacco Tar (from Cigarettes) Introduced into the Lung*

(a) *Smoking machine tar.*—The tar which was prepared by Dr. A. J. Lindsey of the Sir John Cass College from cigarettes in a smoking machine was collected in acetone. The chemical composition of this tar has been reported by Cooper and Lindsey (1955). After evaporating the acetone the tar remaining was dissolved in chloroform and washed in a separating funnel with 1 per cent hydrochloric acid until the supernatant layer showed no nicotine as estimated spectrophotometrically. Thereafter the tar was washed three times with one per cent sodium bicarbonate and twice with distilled water. After removal of the water, the tar (from 200 cigarettes) was suspended in 5 ml. olive oil and of this 0·1 ml. (containing approximately the tar from 4 cigarettes) was injected into the lung. Of the 10 rats inoculated with this amount into the lung, none developed tumours, the earliest death occurring at 320 days and the last at 526, the mean time the animals lived being 476 days (Table III).

TABLE III.—*Intra-pulmonary Injection of Condensate (Tar) from Cigarette Smoke*

| Number of rats | Injection | Result | Lived: Days | Lived: Mean |
|---|---|---|---|---|
| 10 | 0·1 ml. olive oil + tar from 4 cigarettes smoked in smoking machine | No tumour, 10 rats | 320, 393, 438, 488, 510, 513, 520, 526, 526, 526 | 476 |
| 8 | 0·1 ml. olive oil + extract (= 4 cigarettes) from filters + 0·01 mg. killed tubercle bacilli | Sarcoma, 1 rat | 607 | 607 |
| | | Carcinoma, 1 rat | 506 | 506 |
| | | No tumour, 6 rats | 187, 414, 505, 506, 553, 605 | 461 |

(b) *Tar from a proprietary type of filter.*—These filters which can be purchased in Great Britain are of the cartridge type and are fitted into a special holder: they probably contain crystals of silica-gel. About 10 cigarettes of a popular brand were smoked per filter by human subjects and then the filter was replaced by a fresh one. The filters were collected over a period and no special precautions were taken to exclude air from them so that contamination from the air or oxidation of the tar in the filters was possible. The chemical analysis of the extract from these filters has been recorded by Cooper and Lindsey (1955). Twenty filters (through which about 200 cigarettes had been smoked) were broken and extracted in a Soxhlet apparatus with acetone which was later evaporated to leave the residue. The nicotine was removed as described above and the tar suspended in 5 ml. olive oil. To each 1 ml. of this mixture 0·1 mg. of killed tubercle bacilli was added as described in the benzpyrene series under (b). Of this 0·1 ml. containing approximately the tar from 4 cigarettes and 0·01 mg. dead tubercle was injected into the lung.

Eight rats were injected in the lung with the above mixture and lived from 187 to 607 days (mean 485) and of these 2 developed tumours in the lung, one dying 506 days after injection, with a carcinoma, and the other 607 days with a sarcoma. The former was a large whitish tumour which occupied most of the upper lobe (Fig. 18) and there were metastases in the other lobes, particularly around the bronchi. No metastases were found in the liver, kidneys, spleen or suprarenals. Microscopically the large mass in the upper lobe was composed of darkly-staining round and oat-shaped cells (Fig. 19 and 20). The peribronchial infiltration, as noted on naked-eye examination in the lower lobe, also consisted of similar cells (Fig. 21).

The second rat with the sarcoma had a large pinkish growth occupying one lobe (Fig. 22) but no secondary growths were found elsewhere. Histologically the growth was a fibro-sarcoma (Fig. 23), the cells being large, resembling fibroblasts in which fairly numerous mitoses were found. An occasional multinucleated cell was noted but though many sections were examined the spindle-cell character of the growth was maintained throughout. The growth did not spread around the bronchi like the carcinoma but invaded the lung with a solid mass of tumour tissue (Fig. 24).

Into another 4 rats 0·3 ml. of this tar (12 cigarettes), together with 0·05 mg. dead tubercle bacilli, was injected into the thigh muscles. Two of these animals were killed at 506 days and 2 at 517 days after injection. No tumours were present though in all the animals the tar could be recognised in the muscles on naked-eye examination. In the preparation of microscopic sections much of the tar was dissolved, leaving spaces surrounded by fibrous tissue in which there were some foam cells. The persistence of the tar for such a period is of some importance, as commented on later.

### *IV. Controls*

The CB strain of rats has been used for research on tumours for some years at the Chester Beatty Institute in London. Professor A. Haddow, who kindly supplied all the rats necessary for this work, has informed me : " I have had this strain under almost daily observation for many years now and have not observed any spontaneous pulmonary tumours. At the same time, however, very few of the animals we use here have a completely normal life span."

In Table IV the three groups of controls are shown. In the first group the same amount of the same olive oil as was used to suspend the benzpyrene, methylcholanthrene and the smoking machine tar was injected into the lung ; in the second group the same amount of the same olive oil and the same weight of dead tubercle bacilli taken from the same suspension was used for injection as in the experiments with benzpyrene, methylcholanthrene and the tar from the cigarette filters ; in the third group, 2 pellets of the same cholesterol, each weighing 3·83 mg., as was used in the experiments with benzpyrene and methylcholanthrene were inserted into the lung. In each of these control groups, at least half of the animals lived longer than those which developed tumours in the corresponding

TABLE IV.—*Control Experiments*

| Number of rats | Injection into lung | Days lived | Mean days | Control to |
|---|---|---|---|---|
| 4 | 0·15 ml. olive oil | 316, 381, 512*, 512* | 430 | Benzpyrene. Methylcholanthrene. Smoking machine tar. |
| 4 | 0·15 ml. olive oil + 0·01 mg. killed T.B. | 287, 550, 610*, 612* | 515 | Benzpyrene. Methylcholanthrene. Filter tar. |
| 4 | 2 pellets cholesterol = 7·66 mg. | 174, 384, 611*, 612* | 445 | Benzpyrene. Methylcholanthrene. |

* = killed.

benzpyrene, methylcholanthrene or tar groups, but none showed any naked-eye or microscopic evidence of tumour growth in the lungs.

### DISCUSSION

In the CB strain of rats which were employed in this work spontaneous lung tumours are unknown and further in our control rats no growths were observed, though some of these animals lived as long as those treated with known carcinogens or tobacco tar. Therefore, it is safe to assume that any neoplasm which was produced was due to the substances introduced into the lung.

The injection of benzpyrene or methylcholanthrene are known to produce sarcoma when injected into connective tissues. It is not surprising that when these substances were introduced directly into the lungs, which contain a large amount of different types of connective issues, similar to those elsewhere in the body, that sarcomata resulted, particularly with the large amounts which we deliberately employed in these experiments. In the experimental work in animals most cancers have been produced by the direct application of known and suspected carcinogens to tissues such as the skin with a covering of squamous epithelium. In the human lung no such epithelium is normally found, unless as the result of pathological processes such as repeated attacks of acute or sub-acute inflammation, or long continued irritation from inhalation of noxious dusts, smoke or fumes. Under such circumstances the columnar ciliated epithelium of the bronchi may undergo metaplasia to a squamous type, but only as a result of a pathological process of long duration and then in a patchy manner. Auerbach *et al.* (1957) as a result of study of human material found a significant increase in squamous metaplasia of the bronchial epithelium in smokers as compared with non-smokers.

In the CB strain of rats bronchiectasis was noted to be common in the older animals and in them there was patchy de-differentiation of the bronchial epithelium to a lower type, similar to what is observed in the human subject. This was the reason why it was attempted to hasten this change by the inoculation of dead tubercle bacilli together with the known or suspected carcinogens. It was hoped to produce a chronic inflammatory condition which would hasten the de-differentiation of the bronchial epithelium. As the results show, however, the dead tubercle bacilli did not appear to have materially affected the incidence of tumours. It is of some interest that Jensen (1909) originally observed sarcoma in 2 rats which had been inoculated with acid-fast bacilli from a pseudo-tuberculous enteritis of the ox. He does not appear, however, to have considered that there was any relationship between the bacilli and the tumours.

In the benzpyrene series 11 animals developed sarcoma and one carcinoma out of 18 in the group. The mean time taken for the animals to die of sarcoma was 252 days (extremes 159 to 384) and of carcinoma 250 days after inoculation. In the methylcholanthrene series 13 rats out of 18 developed sarcoma and 2 carcinoma. The mean time of death after inoculation for those with sarcoma was 256 days (extremes 134 to 500) and for those with carcinoma 410 days. These times indicate that when known and potent carcinogens are introduced directly into the lung in large amounts, a long interval must elapse before neoplastic change occurs in the lung.

Cholesterol was used as a solvent for benzpyrene and for methylcholanthrene as it allowed of pellets being made that were of such hardness that they could be inserted into the substance of the lung. Though Hieger (1949, 1956) has found that cholesterol even after purification by the Schwenk process had carcinogenic power and Fieser *et al.* (1955) have obtained carcinogens by oxidation of cholesterol, it is unlikely that in view of the potent carcinogens mixed with the cholesterol in our experiments that the cholesterol affected the results. It was observed by Schürch and Winterstein (1935 and 1937) that rabbits fed on a cholesterol-rich diet were more liable to develop warts and cancer as a result of painting the skin with tobacco tar than those which had not been so fed. It may well be that cholesterol deposits act as a solvent for any carcinogen that gains access to the tissues and thus the carcinogen is concentrated at that site. This might in part be the explanation of the high incidence of cancer of the lungs in the older age groups as in them chronic bronchitis is more common, particularly in smokers. It is well known that in chronic inflammatory conditions the occurrence of foam cells, which are rich in cholesterol, is frequent.

None of the rats developed tumours after being inoculated with smoking machine tar, though they survived for a mean time of 476 days (extremes 320 to 526), that is, almost twice as long as the tumour-bearing rats in the benzpyrene and methylcholanthrene series. This tar, however, had been treated with acid to remove the nicotine. The tar from the filters which had been similarly treated, produced 2 tumours but the time interval was long, 506 days after injection for the carcinoma and 607 days for the sarcoma. Cooper and Lindsey (1955) reported that these filters trap only a fraction of the polycyclic hydrocarbons present in the smoke, the proportions being approximately those found in whole smoke. They found that the average 3 : 4 benzpyrene content of the extract from the filters was 1·6 micrograms per 5000 cigarettes. Thus in the tar (from 4 cigarettes) which we introduced into the lungs of rats there was approximately 0·00128 micrograms benzpyrene. Whether this amount would have a carcinogenic effect in the lung is problematical and has yet to be determined. If there is a carcinogen in tobacco tar, it requires to be in contact with the tissues over a long period before a neoplasm results. Wynder, Graham and Croninger (1953) also noted that in mice subjected to painting with the condensate from the smoke from cigarettes that the mean time for the appearance of cutaneous cancer was 71 weeks. This is in accord with the facts as we know them in the case of the human subject where smoking may extend over 30 to 50 years before carcinoma of the lung occurs. It is questionable if any tar which gains access to the tissue would remain chemically and physically unaltered for such a period, though the evidence from some of the experiments where the tar was injected into the muscles seems to indicate, so far as histological appearances go, that this is in fact the case.

In these muscle experiments, however, a large amount of tar was injected at one time whereas in the human subject who inhales the smoke from cigarettes, the tar gains access to the air passages and the lungs in microscopic amounts over a long period. Much of this is probably expelled by ciliary action in the sputum ; that retained, however, must be disposed of by some other mechanism, as, for example, by the growth of epithelium over the phagocytosed particles as described for carbon by Hulse (1955). Tar surrounded by epithelial cells in this manner will be in a most strategic position to exercise any carcinogenic effect on them. It would be of interest if it could be ascertained in experimental animals exposed to the smoke from cigarettes how and where the particles of tar are retained in the pulmonary system.

It might be suggested that the malignant tumours which were produced with tobacco tar from filters were due to contamination from the other known carcinogens with which we were experimenting. This, however, is most unlikely as the experiments with tobacco tar (from the filters) were performed a week before any known carcinogenic hydrocarbon was used. These experiments were carried out in 2 groups of 4 animals each, the rats in each group being injected in the lung one after another with the same suspension of filter tar in olive oil. One group was injected the day before the other and in only one rat in each group did a tumour develop. If any benzpyrene or methylcholanthrene had contaminated the oily suspension of the tar, it is likely that more than one animal in each group would have shown a tumour. Furthermore, all the instruments and syringes which were employed in experiments with known carcinogens were treated in a solution of chromic acid in sulphuric acid to destroy these substances and also the same instruments, syringes, etc., were used, after this treatment, for experiments with the control animals in which no tumours were observed.

### SUMMARY

Experiments are described in which the direct introduction of 3 : 4 benzpyrene, methylcholanthrene and the condensate from cigarette smoke trapped in filters have produced malignant pulmonary tumours (sarcoma and carcinoma) in a strain of rats not subject to the occurrence of spontaneous pulmonary neoplasms.

I wish to record my thanks to Professor A. Haddow for the supply of rats, to Dr. Gillian M. Lewis for preparing some of the carcinogens and the tar from the filters, to Dr. A. J. Lindsey for a supply of cigarette tar from the smoking machine, to Dr. Joan Case for collecting the filters from cigarette smokers, to Mr. G. Brown and Miss Jacqueline Brown for their technical skill and help with the animal experiments and histological preparations, to Mr. P. Crocker for the photographs, to Miss Mauveen Ash for secretarial assistance and to my colleague, Professor Sir Ernest Kennaway, for much kindly criticism.

### REFERENCES

AUERBACH, O., BREWSTER GERE, J., FORMAN, J. B., PETRICK, T.G., SMOLIN, H. J., MUEHSAM, G. E., KASSOUNY, D. Y. AND STOUT, A. P.—(1957) *New Engl. J. Med.* **256**. 97.

COOPER, R. L. AND LINDSEY, A. J.—(1955) *Brit. J. Cancer*, **9**, 304.

ESSENBERG, J. M.—(1952) *Science*, **116**, 561.—(1954) *Ibid.*, **120**, 1000.

FIESER, L. F., GREENE, T. W., BISCHOFF, F., LOPEZ, J. AND RUPP, J. J.—(1955) *J. Amer. chem. Soc.*, **77**, 3928.
FLORY, C. M.—(1941) *Cancer Res.*, **1**, 262.
HIEGER, I.—(1949) *Brit. J. Cancer*, **3**, 123.—(1956) quoted by E. L. Kennaway, *Cancer*, **1**, 24.
HULSE, E. V.—(1955) *J. Path. Bact.*, **69**, 225.
JENSEN, C. O.—(1909) *Z. Krebsforsch.*, **7**, 45.
LAMB, F. W. M. AND SANDERS, E.—(1932) *J. Hyg., Camb.*, **32**, 298.
LORENZ, E., STEWART, H. L., DANIEL, J. H. AND NELSON, C. V.—(1943) *Cancer Res.*, **3**, 123.
PASSEY, R. D.—(1955) *Ann. Rep. Brit. Emp. Cancer Campgn.*, **33**, 60.
ROFFO, A. H.—(1936) *Bol. Inst. Med. exp. Câncer, B. Aires*, **13**, 387.—(1937*a*) *Ibid.*, **15**, 5.—(1937*b*) *Dtsch. med. Wschr.*, **63**, 1267.—(1938) *Bol. Inst. Med. exp. Cáncer, B. Aires*, **15**, 349.—(1939*a*) *Acta Un. int. Cancr.*, **4**, 755.—(1939*b*) *Dtsch. med. Wschr.*, **65**, 963.
SCHÜRCH, O. AND WINTERSTEIN, A.—(1935) *Z. Krebsforsch.*, **42**, 76.—(1937) *Ibid.*, **46**, 414.
SHEAR, M. J.—(1936) *Amer. J. Cancer*, **26**, 322.
WYNDER, E. L., GRAHAM, E. A. AND CRONINGER, A. B.—(1953) *Cancer Res.*, **13**, 855.